# Intake of Red Wine in Different Meals Modulates Oxidized LDL Level, Oxidative and Inflammatory Gene Expression in Healthy People: A Randomized Crossover Trial

**DOI:** 10.1155/2014/681318

**Published:** 2014-04-30

**Authors:** Laura Di Renzo, Alberto Carraro, Roberto Valente, Leonardo Iacopino, Carmen Colica, Antonino De Lorenzo

**Affiliations:** ^1^Section of Clinical Nutrition and Nutrigenomic, Department of Biomedicine and Prevention, University of Rome Tor Vergata, Via Montpellier 1, 00136 Roma, Italy; ^2^CNR, ISN UOS of Pharmacology, Department of Pharmacology, University Magna Graecia, Roccelletta di Borgia, 88021 Catanzaro, Italy; ^3^National Institute for Mediterranean Diet and Nutrigenomics (I.N.Di.M.), Corso Vittorio Emanuele 4, 87032 Amantea, Italy

## Abstract

Several studies have found that adherence to the Mediterranean Diet, including consumption of red wine, is associated with beneficial effects on oxidative and inflammatory conditions. We evaluate the outcome of consumption of a McDonald's Meal (McD) and a Mediterranean Meal (MM), with and without the additive effect of red wine, in order to ascertain whether the addition of the latter has a positive impact on oxidized (ox-) LDL and on expression of oxidative and inflammatory genes. A total of 24 subjects were analyzed for ox-LDL, CAT, GPX1, SOD2, SIRT2, and CCL5 gene expression levels, before and after consumption of the 4 different meal combinations with washout intervals between each meal. When red wine is associated with McD or MM, values of ox-LDL are lowered (*P* < 0.05) and expression of antioxidant genes is increased, while CCL5 expression is decreased (*P* < 0.05). SIRT2 expression after MM and fasting with red wine is significantly correlated with downregulation of CCL5 and upregulation of CAT (*P* < 0.001). GPX1 increased significantly in the comparison between baseline and all conditions with red wine. We highlighted for the first time the positive effect of red wine intake combined with different but widely consumed meal types on ox-LDL and gene expression. *Trial Registration*. This trial is registered with ClinicalTrials.gov NCT01890070.

## 1. Introduction

In the post genomic era, food is considered not only a reservoir of macronutrients, vital in the maintenance of cellular metabolism, but also a major factor capable of determining the quality of health. In fact, the close relationship that exists between micronutrients and gene expression may underlie the pathophysiologic phenomena or, conversely, may represent an early target in delaying the onset of chronic noncommunicable disease (CNCD) [1]. Inflammatory enzymes and oxidative stress are involved in the pathogenesis of numerous inflammatory diseases, including cardiovascular disease (CVD) [2].

The atherosclerotic processes underlying CVD are intimately connected with a state of chronic inflammation involving a variety of pathological changes including endothelial cell activation, low density lipoprotein (LDL) modification, macrophage chemotaxis, and vessel smooth muscle cell migration [3, 4]. Indeed small LDL particles themselves are easily oxidized to yield atherogenic oxidized LDL (ox-LDL) particles, also detectable in healthy subjects [5, 6]. Elevated levels of ox-LDL particles in blood stream have been reported to be associated with increased cardiovascular disease risk [6, 7].

Several epidemiological studies [8–12] that have examined the relationship between the extent of polyphenol-rich food consumption (wine, fruit, tea, and cocoa) and chronic diseases support a protective effect of these antioxidant compounds from cardiovascular disease.

It is widely accepted that the consumption of fruits and vegetables prevents diseases related to the oxidative processes [13]. Several studies have found that adherence to the Mediterranean Diet, due to its unique combination of micro- and macronutrients, appears to have beneficial effects on risk of cardiovascular disease, metabolic syndrome, weight management, several types of cancer, and major chronic degenerative diseases, decreasing overall and cardiovascular mortality [14, 15].

This may be partially mediated through the action of polyphenols present in these foods in their apparent ability to potentiate the endogenous antioxidant system. Mediterranean red wine is an excellent source of polyphenolic compounds such as phenolic acids, flavonoids, stilbenes, and tannins, and a considerable body of research has focused on determining the chemical composition of wine and assaying its* in vitro* antioxidant properties [16].

Different studies have reported positive data on gene expression after feeding animals with phenolic rich extracts or normal food [17, 18]. Rodrigo et al. (2004) demonstrated that superoxide dismutase (SOD), catalase (CAT), and glutathione peroxidase (GPx) activities, all integral to the correct functioning of the antioxidant defense system, were higher in rats after chronic consumption (10 weeks) of red wine as compared to the control group, thus demonstrating the attenuation of oxidative stress by red wine [19]. The processes and enzymatic reactions behind the endogenous response to antioxidant stress have previously been demonstrated [20].

Oxidative stress is closely related to atherosclerotic processes and is believed to be an important secondary consequence of the underlying inflammation which eventually manifests as cardiovascular disease and its complications [21, 22]. Moreover, inflammation is a complex biological process that leads to the coordinated regulation of diverse sets of genes such as chemokine C-C motif ligand 5 (CCL5). CCL5, a chemotactic cytokine (chemokines), usually called RANTES (regulated on activation, normal T cell expresser and secreted), plays diverse roles in the pathology of inflammatory disease [23, 24]. Sirtuins, silent information regulator (SIR), a class of proteins that possess deacetylase or monoribosyltransferase activity, are NAD+-dependent deacetylase regulators of several biological processes such as lifespan, aging, tumorigenesis, neurodegeneration, and metabolic diseases [25]. Seven types of SIRs have been identified in humans [26]. The only cytoplasmic sirtuin protein SIRT2 has been shown to increase in response to oxidative stress but promotes cell death through Forkhead Box proteins (FOXO) [27]. However, the biological function and mechanism of the SIRT2 protein in inflammation and oxidative stress are poorly understood.

In the present study, we evaluated the effect of the consumption of a McDonald Meal (McD) and a Mediterranean Meal (MM) with or without red wine intake on LDL oxidative status. Moreover, we investigated the effects of the two meals on the expression of oxidative stress (SIRT2, SOD, CAT, and GPx) and inflammation (CCL5) genes.

## 2. Materials and Methods

### 2.1. Participants and Study Design

A total of 30 healthy volunteers were recruited by the Clinical Nutrition and Nutrigenomic Section at the University of Rome Tor Vergata. To be eligible for the study, participants had to meet the following inclusion criteria: age between 18 and 65 years and a BMI ≥ 19 Kg/m^2^. Exclusion criteria included active tobacco smoking, arterial hypertension (≥140/90 mm Hg), body mass index (BMI) >30 kg/m^2^, past history of ischaemic coronary artery disease, peripheral or cerebral vasculopathy, hepatic disease, diabetes mellitus, autoimmune disease HIV/AIDS, neoplastic disease, and use of the following medications: NSAIDS, lipid-lowering medications, oral antidiabetic medication or insulin, nitroglycerin, and corticosteroids.

At baseline, all participants were evaluated in terms of their health status. The clinical evaluation focused on nutritional status, blood pressure, clinical-biochemical analysis, quantification of ox-LDL, and a genomic evaluation with analysis of five genes belonging to the pathway of oxidative stress and inflammation.

**Figure 1:**
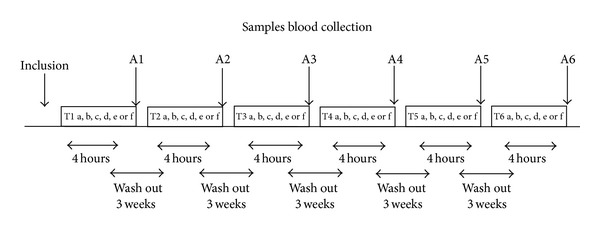
Study design and diets. This randomized crossover study was divided into six treatment interventions (T1, T2, T3, T4, T5, and T6) each lasting 4 hours, split by three 3-week washout periods with total study period of 18 weeks. In each treatment period (T1, T2, T3, T4, T5, and T6), volunteers consumed (a) baseline (fasting); (b) fasting + 250 mL red wine (FRW); (c) Mediterranean Meal (MM); (d) MM + 250 mL red wine (MMRW); (e) McDonald's Meal (McD); (f) McD + 250 mL red wine. The oxidative status of each volunteer was evaluated at baseline and at the end (A1, A2, A3, A4, A5, and A6) of each treatment period.

The experimental study was conducted according to a randomized crossover trial with six arms (T1, T2, T3, T4, T5, and T6), as shown in the diagram presented in Figure 1.

During the study period volunteers consumed in a randomized order (a) baseline (B); (b) fasting + 250 mL red wine (FRW); (c) Mediterranean Meal (MM) [13] (carbohydrates 55–60% of total Kcal; protein 15–20% of total Kcal of which 50% are of vegetable derivation; total fats <30% of total Kcal; saturated fat <10% of total Kcal; polyunsaturated fatty acids (PUFA) 6–10% of total Kcal: 5-6% of total Kcal from n-6 PUFA, and 1-2% of total Kcal from n-3 PUFA; monounsaturated fatty acids (MUFA) about 15% of total Kcal; trans-fatty acids <1% of total Kcal; 30 g of fiber); (d) MM + 250 mL red wine (MMRW); (e) McDonald's Meal (McD) (n.1 sandwich Big Tasty Bacon and n.1 small French Fries package: carbohydrates 26.8% of total Kcal; protein, 18.2% of total Kcal (of which about 70% was comprised of animal proteins); total fat 55% of total Kcal McD; (f) McD + 250 mL red wine (McDRW).

Each intervention was followed by a 3-week washout period to avoid additive effects on treatments to follow.

The parameters of body composition were collected at baseline. Samples for the genomic and biochemical analysis were collected at baseline and 4 hours after each meal intervention.

Participants were not blinded to the type of diet they consumed.

The MM was prepared and distributed by the staff of the Clinical Nutrition and Nutrigenomic Section, Department of Biomedicine and Prevention, University of Rome Tor Vergata.

Subjects were asked to maintain their usual lifestyle habits and to report any illness or abnormality presented during the study period. At the end of each arm, a clinician assessed any adverse effects from the interventions by going through a checklist of symptoms including bloating, fullness, or indigestion, altered bowel habit, dizziness, and other symptoms that were possibly associated with the interventions. All patients completed the study.

Nutritional status assessment and genomic analysis were performed at the Clinical Nutrition and Nutrigenomic Section, Department of Biomedicine and Prevention of University of Rome Tor Vergata.

### 2.2. Anthropometric Measurements

After a 12 h overnight fast, all subjects underwent anthropometric evaluation. Anthropometric parameters of all the participants were measured according to standard methods (body weight, height, and waist and hip circumferences) [28]. Subjects were instructed to take off their clothes and shoes before performing all the measurements. Body weight (Kg) was measured to the nearest 0.1 Kg, using a balance scale (Invernizzi, Rome, Italy). Height (cm) was measured using a stadiometer to the nearest 0.1 cm (Invernizzi, Rome, Italy). The waist (WC) and hip (HC) circumferences were measured with a flexible steel metric tape to the nearest 0.5 cm. WC was measured on the horizontal plane that corresponds with the narrowest point between the iliac crest and the bottom rib. HC was measured at the largest point when observed on a horizontal plane. BMI was calculated using the formula BMI = body weight (Kg)/height (m)^2^.

The blood pressure was taken using a mercury sphygmomanometer on the right upper arm after the subject was seated quietly for at least 5 min (average of three measurements).

### 2.3. Bioelectrical Impedance Analysis (BIA)

Resistance (*R*), reactance (*X*_*c*_), impedance, and phase angle (PA) at 50 kHz frequency (single frequency, SF) were measured using BIA phase-sensitive system (BIA 101S, Akern/RJL Systems, Florence, Italy). Measurements were taken on the left side of the body with injection and sensor electrodes placed on the hand and foot in the reference position. TBW, extracellular water (ECW), intracellular water (ICW), Na/K ratio, PA, body cell mass (BCM), and body cell mass index (BCMI) were calculated from bioelectrical measurements and anthropometric data by applying the software provided by the manufacturer which incorporated validated predictive equations [29].

### 2.4. Dual-Energy X-Ray Absorptiometry (DXA)

Body composition analysis was assessed by DXA (DXA, GE Medical Systems, Milwaukee, WI, USA) according to the previously described procedure for evaluating soft tissues, that is, TBFat and TBLean [29, 30]. The subjects were instructed not to exercise within 24 h of the test. The subjects were given complete instructions on the testing procedure. They wore a standard cotton t-shirt, shorts, and socks. They lay supine on the DXA scanner without moving for the duration of the scan. The average measurement time was 20 min. Radiation exposure was equivalent to 0.01 mSv. The intra- and intersubject coefficient of variation (CV% = 100 × s.d./mean) ranged from 1 to 5%. The coefficient of variation for bone mass measurements was ≤1%; the coefficients on this instrument for five subjects scanned six times over a 9-month period were 2.2% for TBFat and 1.1% for TBLean.

### 2.5. Sample Collection and RNA Extraction

A fasting blood sample was collected and stabilized in PAXgene Blood RNA Tubes (PreAnalytiX Qiagen, Hombrechtikon, Switzerland) and stored at −80°C until RNA extraction. The total RNA of each collected sample was purified using the PAXgene Blood miRNA Kit according to the manufacturer's instructions (PreAnalytix Qiagen, Hombrechtikon, Switzerland). Aliquots of total RNA were then quantified and assessed for quality by spectrophotometry (Nanodrop, Wilmington, USA) and agarose gel electrophoresis.

### 2.6. Quantitative Real Time PCR and Data Analysis

We used specific RT^2^ Profiler PCR Arrays (Qiagen, Netherlands); for our study we focused on the Human Oxidative Stress (PAHS-065ZA) pathway, in particular SIRT2 CCL5SOD, CAT, and GPx. Each qRT-PCR experiment was performed in triplicate and repeated at least twice according to the manufacturer's instructions (Qiagen, Netherlands). The comparative threshold (CT) cycle was used to determine the gene expression level relative to the calibrator RNA from the controls. Steady state mRNA levels were expressed relative to the calibrator as “*n*-fold” differences. The CT value for each gene was normalized using the formula ΔCT = CT (*gene*)-CT (*housekeeping gene*).

In particular we used the average of 4 housekeeping genes included in the plates actin beta* ACTB* (NM_001101), hypoxanthine phosphoribosyltransferase, HPRT1(NM_000194), beta-2-microglobulin, BM2 (NM_00404080), and glyceraldehyde-3-phosphate dehydrogenase, GAPDH (NM_002046). The relative gene expression levels were determined according to the following formula: ΔΔCT = ΔCT sample −ΔCT calibrator.

The value used to plot relative gene expression was determined using the expression fold change (FC) = 2^−ΔΔCT^. Raw data were filtered for genes that were significantly changed above factor 1.0 within the 95% confidence interval (*P* ≤ 0.05) for each experiment. Finally, only genes with an absolute FC value of at least ±1.5 and *P* value ≤ 0.05 (indicating a statistical significance) were considered as differentially expressed genes.

### 2.7. Low Density Lipoprotein Oxidative Status

Blood samples were collected and stabilized in EDTA. Analysis of the level of oxidation of the organism was observed by the quantification of protein and oxidized LDL from the nutrigenomic study. An ELISA test was utilized for the study of LDL using the Mercodia oxidized LDL ELISA (Mercodia Diagnostic, Sweden) according to the customer protocol.

#### 2.7.1. Red Wine Description

Masieri red wine (by Biancara of Angiolino & Alessandro Maule, Gambellara, Italy, 2012) was used in the study. This wine is made from a selection of mixed grapes including Merlot (75%), Tocai Rosso (10%), and Cabernet Sauvignon (15%) grown in volcanic soils using natural methods and is produced using the spontaneous fermentation method. The wine's characteristics are as follows: unfiltered wine, without added sulphites; total alcohol: 14.52% volume; relative density: 0.9 g/L; residual sugar: 0.7 g/L; total acidity: 5.9 g/L; dry extract: 30 g/L; volatile acidity: 0.59 g/L; total sulfur dioxide: 2 mg/L.

### 2.8. Statistical Analysis

A paired *t*-test or a nonparametric Wilcoxon test was performed to evaluate differences before and after nutritional intervention. All tests were considered significant at *P* ≤ 0.05. Statistical analysis was performed using a computer software package SAS version 9.3 (SAS Institute, Cary, NC).

## 3. Results

### 3.1. Clinical Trial

Of the 30 initial participants initially enrolled, 24 subjects were eligible for the study. Three subjects declined to participate during the first phase, and another three did not meet the inclusion criteria: one of them measured a BMI < 19 Kg/m^2^, one had diabetes mellitus, and one had a previous history of ischemic heart disease.

Table 1 shows the baseline characteristics of all 24 individuals. No subjects were obese on the basis of BMI classification; however 25% of the subjects were overweight. On the contrary, 25% of subjects had a PBF% > 35 and were therefore classified as obese. None of the subjects were osteoporotic, and, according to ASMMI, 4% of subjects were sarcopenic. No subjects were hypertensive.

**Figure 2:**
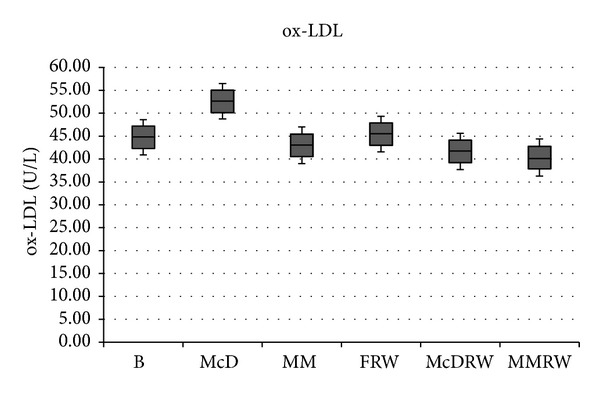
Oxidized LDL level. Comparative values of ox-LDL level for each treatment intervention. B: baseline; McD: McDonald Meal; MM: Mediterranean Meal; FRW: fasting + 250 mL red wine; McD RW: McDonald Meal + 250 mL red wine; MMRW: Mediterranean Meal + 250 mL red wine. The significant values are expressed as (a) *P* ≤ 0.05 and (b) *P* > 0.05: (a) B versus McD; McD versus MM; FRW versus MMRW; McD versus McDRW and (b) B versus FRW; B versus MM; MM versus MMRW; FRW versus McD RW; McD RW versus MMRW; B versus McDRW; B versus MMRW.

The comparison of ox-LDL level in the intervention treatments was shown in Figure 2. A significant increase (*P* ≤ 0,05) of ox-LDL in the B compared to McD (Δ% = 17.5%) was highlighted.

Ox-LDL levels significantly decreased (*P* ≤ 0.05) under the following conditions: (i) McDM versus MM (Δ% = 18.2%); (ii) FRW versus MMRW (Δ% = 11.3%); (iii) McD versus McDRW (%Δ = 20.78%). No significant differences (*P* > 0.05) in the level of ox-LDL were observed under the following conditions: (i) B versus FRW (Δ% = 1.59%); (ii) B versus MM (Δ% = 3.87%); (iii) MM versus MMRW (Δ% = 6.23%); (iv) FRW versus McDRW (Δ% = 8.32%, *P* > 0.05); (v) McDRW versus MMRW (Δ% = 3.22%); (vi) B versus McDRW (Δ% = 6.86%); (vii) B versus MMRW (Δ% = 9.85%).

**Figure 3:**
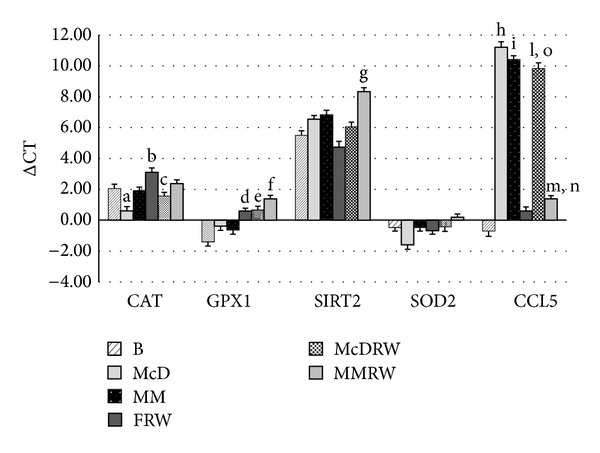
Variation in gene expression following each intervention. ΔCT value of gene expression of CAT, GPX1, SIRT2, SOD2, and CCL5 under the different conditions analyzed after each treatment intervention. B: baseline; MM: Mediterranean Meal; McD: McDonald Meal; MMRW: Mediterranean Meal with red wine; McDRW: McDonald Meal with red wine; CAT: catalase; GPX1: glutathione peroxidase 1; SOD2: superoxide dismutase 2; SIRT2: sirtuin 2; CCL5: chemokine ligand 5. The significant values are expressed as *P* ≤ 0.05: (a)  ΔCT value of gene expression of CAT: B versus McD; (b) ΔCT value of gene expression of CAT: B versus FRW; (c)  ΔCT value of gene expression of CAT: McD versus McDRW; (d) ΔCT value of gene expression of GPX1: B versus FRW; (e)  ΔCT value of gene expression of GPX1: B versus McDRW; (f)  ΔCT value of gene expression of GPX1: B versus MMRW; (g) ΔCT value of gene expression of SIRT2: FRW versus MMRW; (h) ΔCT value of gene expression of CCL5: B versus McD; (i) ΔCT value of gene expression of CCL5: B versus MM; (l) ΔCT value of gene expression of CCL5: B versus McDRW; (m) ΔCT value of gene expression of CCL5: B versus MMRW; (n) ΔCT value of gene expression of CCL5: MM versus MMRW; (o) ΔCT value of gene expression of CCL5: FRW versus McDRW.

Moreover, we analyzed the variation of gene expression of five genes related to oxidative stress and inflammation depending on consumption of different meals with and without red wine (Figure 3).

CAT expression decreased significantly (*P* ≤ 0.05) after McD. On the contrary, a significant increase (*P* ≤ 0.05) of CAT expression was observed between B versus FRW and between McD versus McDRW.

GPX1 expression increased significantly (*P* ≤ 0.05) in the comparison between (i) B versus FRW; (ii) B versus McDRW; and (iii) B versus MMRW.

SIRT2 expression increased significantly (*P* ≤ 0.05) in comparison of FRW versus MMRW. No significant SOD expression was observed in all conditions.

CCL5 expression significantly increased (*P* ≤ 0.05) in the comparison between (i) B versus McD; (ii) B versus MM; (iii) B versusboth meals with wine (MMRW and McDRW); and (iv) FRW versus McDRW. Meanwhile, CCL5 expression significantly decreased (*P* ≤ 0.05) between MM versus MMRW.

The value of the Pearson coefficient of *R* = 0.89 shows a positive correlation (*P* < 0.001) between SIRT2 and CAT expression in McD and MMRW. The value of the Pearson coefficient of *R* = −0.91 shows a negative correlation (*P* < 0.001) between expression of SIRT2 and CCL5 in MM and McD.

## 4. Discussion

Interactions between genetic and environmental factors such as diet and lifestyle, particularly in the case of overnutrition and sedentary behavior, promote the progression and pathogenesis of polygenic diet-related diseases, the prevalence of which is increasing to epidemic proportions.

The effects of dietary compounds on metabolic pathways related to cardiovascular diseases, diabetes, and other CNCD are currently under investigation and are leading the traditional methods of nutritional counseling towards a more complex approach based on the modulation of gene expression by food.

The evidence connecting nutritional factors to the etiology of cardiovascular disorders is compelling [31]. CVDs have multiple causes, but the majority of CVD events originate from the complications of atherosclerosis, a pathophysiological process that can be prevented by nutritional interventions [32]. For a long time, based on the results of experimental studies carried out* in vitro*, the preventive effect of phenolics on age-related chronic diseases, such as CVD, was attributed to their antioxidant capacity [33]. The existing data indicate that the role of fruits and their associated nutrients in cardiovascular prevention may be more influential than that of vegetables alone; however, due to the disappointing results of a number of large interventional studies performed with these micronutrients, showing no reduction in overall mortality and even an increased cardiovascular risk, scientists were led to consider other potentially beneficial compounds present in fruits and vegetables [12, 34, 35]. Many studies have emphasized their ability to protect various cellular constituents against oxidation [36].

Among many genetic and environmental causes, the accumulation of modified LDL [37], such as ox-LDL, and recruitment of monocyte-derived macrophages at the atrial subendothelial space [38] are the key factors leading to the development of an atherosclerotic lesion [39]. Indeed, small LDL particles are easily oxidized to yield the atherogenic ox-LDL particles that can accumulate in the foam cells of the atherosclerotic plaque [40].

Antioxidant flavonoids and polyphenols became the first substances present in red wine shown to have proven beneficial effects in various diseases, such as inhibition of LDL oxidation or attenuation of ischemia-reperfusion injury [41, 42]. Red wine exhibits higher antioxidant capacity and protective effect against LDL oxidation when compared to white wine [43–45].

We observed a significant reduction of ox-LDL depending on of the quality of meal consumed. In particular the values for ox-LDL were significantly decreased (*P* ≤ 0.05) after the MM alone; MMRW increases the protective effect (*P* ≤ 0.05). On the other hand, the consumption of McD increases the values of ox-LDL (*P* ≤ 0.05), while McDRW brings the values for ox-LDL back towards baseline levels (*P* > 0.05).

Our findings correspond with the observation that moderate alcohol consumption, in particular red wine, is associated with a reduced risk for cardiovascular disease and an improved lipid profile [46–51]. In agreement with Tomè-Carneiro et al. (2012) [52] we observed that the consumption of red wine significantly decreases the level of ox-LDL (*P* ≤ 0.05), resulting in a more efficient endogenous antioxidant defense system, with the better protection from oxidative and inflammatory damage [53]. Polyphenols appear to interact with molecular signaling pathways and related cellular machinery that regulate processes such as inflammation and consequently oxidative status [54].

We investigate, in human peripheral blood mononuclear cells (PBMC), the expression of genes related to antioxidant defenses (SIRT2, SOD, CAT, and GPx) and inflammation (CCL5) after consumption of MM and McD with or without red wine.

Particular attention was given to superoxide dismutase (SOD), catalase (CAT), and glutathione peroxidase 1 (GPX1) for their fundamental role in reducing intracellular reactive oxygen species (ROS) levels, thus protecting against cell/tissue damage [53, 55, 56]. We found that SOD expression did not significantly differ between intervention arms in our study, in agreement with Rodrigo et al. (2002) [18]. However, CAT expression was upregulated after MM, MMRW, and McDRW, probably due to the enrichment of the meals with antioxidant compounds.

SIRT2 modulates ROS production and increases resistance to its damaging effects [57, 58]. It is interesting to observe the different levels of SIRT2 expression after consumption of the MM and McD, with and without red wine, likely attributable to amplified expression of SIRT2 in response to the resveratrol present in red wine, as suggested by Lagouge et al. (2006), Schirmer et al. (2012), and Mukherjee et al. (2009) [58–60].

After McD and McDRW consumption, SIRT2 is expressed to a lesser extent than after MM, in agreement with Kim et al. (2013), where SIRT2 is upregulated in response to calorie restriction and oxidative stress, and promotes cell death under severe stress conditions via interaction with FOXO3a [56].

We observed a higher expression of SIRT2, after MM with red wine, which was negatively correlated (*P* < 0.001) with expression of CCL5, an important chemokine involved in inflammatory process. This result is in accordance with Lin et al. (2000) [61], who observed a protective effect of transduced PEP-1-SIRT2 against inflammation and oxidative stress in murine macrophages. Our results are probably due to the antioxidant characteristics of this meal in association with the polyphenols of red wine, possibly representing an optimal nutritional combination. On the other hand, lower expression of SIRT2 in the McD is correlated (*P* < 0.001) with a high level of CCL5 expression. Lin et al. (2000) [61] and Zheng et al. [62] suggested that antioxidants effectively suppressed CCL5 mRNA expression, indicating that oxidation may be involved in the induction of the CCL5 gene expression by dengue-2-virus infection. In our results, CCL5 expression is lower after MMRW consumption, which attests to the proposed antioxidant and anti-inflammatory activity of red wine and food polyphenols.

A positive correlation (*P* < 0.001) between SIRT2 and CAT was observed, in McD and MMRW, which may be due to SIRT2 increasing expression of CAT [62]. To summarize, modulation of SIRT2 through diet may have a significant impact on the inflammation underlying chronic noncommunicable diseases.

On the other hand, consumption of a McD was related to prooxidant and proinflammatory activity, as demonstrated by the increase of LDL oxidation and hyperexpression of the inflammatory CCL5 gene. Interestingly, associating red wine with the McD attenuates this effect.

## 5. Conclusion

The effect of red wine in association with McDonald's and a Mediterranean Meal on ox-LDL and gene expression was studied for the first time, with positive results indicating that the antioxidant potential of the nutrients found in red wine and the Mediterranean Diet may be an essential component of a holistic approach to combatting chronic noncommunicable diseases linked to inflammation.

However, prospective long-term data on consumption of a Mediterranean Diet as opposed to Western Diet (with or without red wine) on ox-LDL and gene expression is not yet available and deserves further research in order to verify changes on body composition related to gene expression. Although the number of subjects enrolled in our study is acceptable in this instance [1], much greater numbers are required to definitively confirm these results.

In conclusion, this study provides an interesting insight into the possibility of preventing future illness through manipulation of environmental factors including diet, in line with the concept of “prospective health care,” according to predictive, preventive, and personalized medicine [63].

## Figures and Tables

**Table 1:** Baseline characteristics of healthy volunteers.

Parameters	Median ± SE	Min–max
		(*n* = 15)
Age (y)	31,04 ± 5,88	25,00–46,00
SBP (mmHg)	110,22 ± 11,42	100,00–130,00
DBP (mmHg)	70,67 ± 8,83	60,00–88,00
Height (cm)	168,92 ± 10,03	157,00–183,00
Weight (Kg)	65,12 ± 9,85	48,00–79,00
BMI (Kg/m^2^)	23,24 ± 2,32	20,00–27,10
WC (cm)	75,64 ± 5,59	68,00–88,00
HC (cm)	97,23 ± 6,44	88,00–108,00
*W*/*H*	0,78 ± 0,07	0,67–0,88
TW (%)	53,94 ± 3,80	47,80–59,70
IW (%)	44,35 ± 3,93	39,50–51,20
EW (%)	55,74 ± 4,00	48,80–60,50
BCMI	9,45 ± 1,28	7,80–11,80
BMD (g/cm^2^)	1,14 ± 0,11	0,95–1,34
*T*-score	0,12 ± 0,91	−1,30–1,40
BMC (Kg)	2,57 ± 0,54	1,90–3,80
PBF (%)	28,79 ± 6,36	18,80–40,90
TBF (Kg)	18,58 ± 4,56	11,71–27,97
Lean arms (Kg)	5,02 ± 1,24	3,39–6,96
Lean legs (Kg)	14,57 ± 3,38	9,81–19,65
TBL (Kg)	43,80 ± 8,53	31,54–57,09
RMR (Kcal)	1517,69 ± 194,17	1175,00–1805,00
ASMMI (kg/m^2^)	6,78 ± 0,97	5,33–8,35

Baseline characteristics of subjects at the beginning of the study. Results are expressed as median ± standard error and minimum (min) and maximum (max) for each parameter.

SBP: systolic blood pressure; DBP: diastolic blood pressure; BMI: body mass index; WC: waist circumference; HC: hip circumference; *W*/*H*: waist hip ratio; TW: total water; IW: intracellular water; EW: extracellular water; BCMI: body cellular mass index; BMD: bone mineral density; BMC: bone mineral content; PBF: percentage of total body fat mass; TBFat: total body fat mass; TBLean: total body lean mass; RMR: resting metabolic rate; ASMMI: appendicular skeletal muscle mass index.
